# Pharmacist assisted vape taper and behavioral support for cessation of electronic nicotine delivery system use

**DOI:** 10.1002/ccr3.2599

**Published:** 2019-12-08

**Authors:** Michelle Sahr, Shelby E. Kelsh, Noah Blower

**Affiliations:** ^1^ College of Pharmacy Ferris State University Big Rapids MI USA

**Keywords:** cessation, electronic nicotine delivery systems, nicotine taper, vaping

## Abstract

Tapering of vaping, reducing nicotine concentration and restricting vaping times, coupled with behavioral counseling may be effective for cessation of electronic nicotine delivery system use.

## Graphical abstract

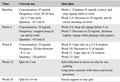

## 1 INTRODUCTION

Electronic nicotine delivery systems (ENDS) have gained popularity in recent years. Despite increased use, there is a clear gap in knowledge related to cessation of ENDS. ENDS have been used as a cessation aid for cigarettes, with various studies reaching different conclusions.^1^, ^2^, ^3^ A growing concern is that many people who switch to ENDS continue to use their ENDS long term.^3^ Additionally, there is no published research on how to help users quit besides one case report using nicotine replacement therapy.^4^ We describe our experience with an alternative approach to cessation of ENDS through an ENDS use taper and behavioral counseling.

## 2 CASE DESCRIPTION

A 23‐year‐old male who vapes daily enrolled in a vaping cessation pilot program. He was motivated to quit due to a decrease in energy and difficulty exercising. His only medical conditions were a slight hearing impairment and self‐reported acid reflux. He did not take any prescription or nonprescription medications. At baseline, his blood pressure was 133/82 mm Hg, and he weighed 91 kg. He had a 6‐year social history of smoking cigarettes and attempted to quit nicotine products six times previously. During the first 4 years, he would typically smoke friend's cigarettes. This escalated to smoking at least two cigarettes daily. He switched from cigarettes to vaping, when he noticed constant cold. He has been vaping daily for 18 months. During that time, he switched devices and concentrations frequently. Initially, when vaping, he used the lowest nicotine concentration he could find, reportedly 5 mg/mL. He switched to a smaller device after discovering the bigger devices burned through e‐juice fast. However, his new device required e‐juice concentration of 25 mg/mL or higher, so he increased his nicotine concentration. When he tried to quit vaping in the past, stress and social environment drove him to continue using nicotine products. Using a modified Fagerstrom Test for Nicotine Dependence (FTND), he rated a high dependence on nicotine at his initial appointment (Table 1). Additional social history included consuming roughly 2‐3 alcoholic beverages per week and past marijuana use.

**Table 1:** Modified Fagerstrom test for nicotine dependence with scoring

Question	Score	Day 1	4 wk	8 wk	12 wk	24 wk
How soon after waking do you vape?	<5 min (3 pt) 5‐30 min (2 pt) 31‐60 min (1 pt) >60 min (0 pt)	3	1	0	0	0
Do you find it difficult to refrain from vaping in places where it is forbidden?^a^	Yes (1 pt) No (0 pt)	1	0	0	0	0
Which vaping would you hate to give up?	First in the morning (1 pt) Other time (0 pt)	0	0	0	0	0
How many times a day do you vape?^b^	0‐10 (0 pt) 11‐20 (1 pt) 21‐30 (2 pt) >30 (3 pt)	3	1	0	0	0
Do you vape more frequently in the morning?	Yes (1 pt) No (0 pt)	0	0	0	0	0
Do you vape even if you are sick in bed most of the day?	Yes (1 pt) No (0 pt)	1	1	0	0	0
Total score^c^		8	3	0	0	0

a Forbidden locations: church, class, airplanes, etc

b One time = 5 min.

c Dependence: low (0‐2 pt), low/moderate (3‐4 pts), moderate (5‐7 pts), high (8‐10 pts).

**Figure 1:**
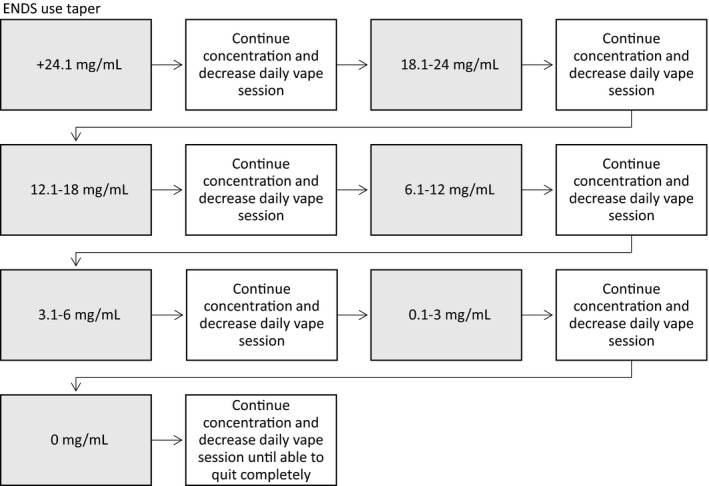
Electronic nicotine delivery systems (ENDS) use taper. Start at the patient's current e‐juice nicotine concentration. The taper will aim to decrease amount of nicotine consumed by reducing concentration and frequency over time. The first week the patient will decrease vaping by one session per day or decrease duration of sessions. The next week the patient will decrease e‐juice nicotine concentration. If unable to complete the step, then repeat the same step until successful before moving on. Steps will be followed until patient has stopped vaping and is nicotine free

A pharmacist guided the participant through a quit attempt using behavioral support and a taper of both nicotine e‐juice concentration and vaping sessions. The taper was created to help patients stop using ENDS and be nicotine free within 12 weeks of enrollment (Figure 1). He used his own Suorin Air™ ENDS and 45 mg/mL watermelon flavored nicotine e‐juice. At baseline, he reported vaping frequently (every 20‐30 minutes) for 2‐3 minutes per session and would vape approximately 5 mL a week. A quit plan was created at each appointment focusing on the upcoming 4 weeks (Table 2). He was using a higher nicotine concentration than the ENDS taper (Figure 1) was designed for, so the same methodology was used until using a lower concentration. The first step was to continue using his current 45 mg/mL e‐juice and stop vaping during work, 9:00 am to 12:00 pm This time was chosen, as he believed it would be the easiest time to quit. For his next 3 weeks, he was to purchase his preferred brand's next lowest concentration (35 mg/mL) nicotine e‐juice and continue to decrease morning sessions.

**Table 2:** Quit attempt summary

Time	Current use	Quit plan
Baseline	Concentration: 45 mg/mL Frequency: every 20‐30 min for 2‐3 min each Quantity: ~0.7 mL/d	Week 1: Continue 45 mg/mL e‐juice, and stop vaping while at work Week 2‐4: Decrease to 35 mg/mL and decrease morning sessions
Week 4	Concentration: 35 mg/mL Frequency: stopped using in car and at work Quantity: ~0.5 mL/d	Week 5‐6: Stop all vaping before 5 pm Week 7: Decrease to 25 mg/mL, decrease nightly vaping while playing video games.
Week 8	Concentration: 25 mg/mL Frequency: 20 hits between 8‐11 pm Quantity: ~20 puffs/d	Week 9: Vape only in a 2‐3 h window Week 10: Decrease to 5‐10 mg/mL Week 11: Vape only in 1 h window Week 12: Quit
Week 12	Quit for 2 wk	Self‐reflection to focus on why he was quitting Long term concerns with stress and social pressures
Week 24	Quit for 14 wk	Social support to stay quit

At his follow‐up call 5 days after his initial appointment, he was using 45 mg/mL e‐juice, and he had decreased the frequency of his morning vaping. He had not experienced any changes in mood, but he did notice that he was bouncing his leg more often. He also noted better sleep and that his acid reflux, which was better on ENDS than cigarettes, was improving with decreased use. He found that setting time constraints on his vaping were helpful, as well as keeping his ENDS device out of sight/reach to make vaping less accessible. The biggest obstacle he foresaw in his quit attempt was absentmindedly vaping out of habit.

By his next appointment at 4 weeks, he had successfully decreased e‐juice concentration to 35 mg/mL and had cut out vaping sessions in the car and at work. He felt more ornery than normal, but his restlessness had subsided by the end of the second week. He reiterated that he was sleeping better, and indigestion had improved. He affirmed that keeping his ENDS out of sight and reach was still helping him reduce sessions, but this time he felt social situations remained his biggest challenge in the quit attempt. He stated that his close friends were helping support him and would call him out when he tried to vape in order to combat this. His modified FTND score at week 4 had decreased to 3, indicating that his nicotine dependence was now moderate to low. His quit plan over the next 4 weeks was to stop all vaping before 5 pm and continue not vaping at work, school, or in the car. By week 7, his goal was to decrease his nicotine concentration to 25 mg/mL.

At his 6‐week phone call follow‐up, the individual had decreased his e‐juice to a 25 mg/mL nicotine concentration. He had also successfully cut out all vaping before 5:00 pm Since baseline, he decreased his usage by half and was now using <2.5 mL of e‐juice weekly. He did report thinking about vaping more when he decreased e‐juice concentration, but kept his ENDS out of sight and focused on why he was decreasing his vaping helped him be successful.

At his 8‐week follow‐up appointment, he had again decreased the frequency of his vaping and was now only vaping between 8 and 11 pm (~20 puffs total). His modified FTND score was now 0 indicating low dependence. He was very happy with his quit attempt and felt that it was very rewarding. He again noted that keeping his ENDS out of sight was the biggest factor in his success so far. In his 9th week, his goal was to cut down his vaping to a 2‐3 hour window. For his 10th week, he was to decrease e‐juice concentration to 5‐10 mg/mL. By his 11th week, he was to vape only during a 1 hour window. For his 12th week, his goal was to stop vaping and be completely nicotine free.

At his 10‐week follow‐up call, the individual noted that he could not find a lower concentration of e‐juice in the brand he used, so he quit vaping completely. He found that he was more irritable and struggled to concentrate at night when he would usually vape. He had begun to practice self‐reflection to focus on why he was quitting which helped to overcome this. He also reported coughing less since quitting.

At his 12‐week final appointment, he was still not vaping or using nicotine. His blood pressure was now at a normal range of 115/69 mm Hg, and weight remained unchanged. No additional side effects of withdrawals were noted, and his modified FTND score was 0. He realized stress and social pressures will remain, but is hopeful that this will not interfere with his successful quit.

At the patient's 6‐month follow‐up call, he stated that he was not using any nicotine or vaping products. He reported, “I had a couple slip ups at a wedding and a few other times (alcohol was a key factor in all honesty), but when I'm with supportive friends who don't allow me to continue the habit‐I have no issues.”

## 3 DISCUSSION

We believe this is the first case report describing a successful ENDS quit attempt using an ENDS taper with motivational interviewing. There are multiple pieces that are unique in this report. First, the creation of a taper which aligns with many ENDS users personal plans for quitting (Figure 1). This taper was designed to slowly wean users off to avoid withdrawal symptoms. The key to this taper is to alternate between decreasing nicotine concentration and ENDS use. If you only decrease one, then the patient can alter their vaping to maintain their nicotine exposure. The additional benefit of using a taper instead of traditional nicotine replacement products is the ability to gradually work toward a quit date which leads to more patient willingness to engage with a quit attempt. There is the risk of continuous exposure to e‐juice liquid, so risks and benefits must be weighed by the clinician and patient. We also modified the FTND score to vaping to estimate nicotine dependence. While the tool is not validated, there was a trend to decreasing nicotine dependence over the taper. There are needed modifications since vaping is more challenging to quantify than cigarettes, the strong urge to smoke is the morning is not the same with vaping, and there are fewer restrictions on where vaping can occur. The authors are not aware of any validated tools to assess vaping dependence and did not want this to delay helping users to quit.

A key take away for clinicians is learning barriers to ENDS cessation and adjusting to individual patient needs. An obstacle our patient faced was absentminded ENDS use. It does not have the same rituals or sessions as smoking a cigarette does, so it is very easy for patients to use without even thinking about it. This is likely related to the fact that there are fewer restrictions on the use of ENDS in public compared with cigarettes which enable people to use in more locations and more frequently. Also, ENDS are socially acceptable, and in some patient populations, like college students, it is very common. We focused our counseling on increasing self‐awareness and improving skills needed to overcome the addiction to nicotine and the habit of vaping. Having a week‐by‐week plan for the patient allowed him to have clear goals, and frequent check‐ins kept him accountable. Addiction is a lifelong factor, so patients should understand that cravings will occur and slipups may happen, but it is imperative to focus on the skills developed to stay strong. If a patient does have slipups, healthcare practitioners should encourage patients to keep working toward their end goal of staying nicotine free, but be supportive that this is a challenging disease to face.

## 4 CONCLUSION

A vaping tapering schedule coupled with behavioral support can be an effective quitting technique. When a tapering schedule is used, pairing decreased nicotine concentration with decreased time spent vaping seems to be highly effective. One method to decrease time spent vaping is to work at eliminating vape sessions by 10%‐15% on weeks, when e‐juice nicotine concentration remains unchanged. A key to decreasing sessions seems to be decreasing mindless vaping. It is vital for healthcare professionals to aid users in quitting ENDS and reinforce the goal of quitting vaping and being nicotine free.

## CONFLICT OF INTEREST

None declared.

## AUTHOR CONTRIBUTIONS

MS: provided clinical care to patient, collected data, was involved in manuscript drafting and editing, and approved final version. SK: provided clinical care to patient, collected data, was involved in manuscript drafting and editing, and approved final version. NB: collected data, was involved in manuscript drafting, and approved final version.
